# Metastatic melanoma to small bowel: metastasectomy is supported in the era of immunotherapy and checkpoint inhibitors

**DOI:** 10.1186/s12957-024-03335-3

**Published:** 2024-03-11

**Authors:** Paul Wong, Andrew D. Wisneski, Katy K. Tsai, Tammy T. Chang, Kenzo Hirose, Eric K. Nakakura, Adil I. Daud, Ajay V. Maker, Carlos U. Corvera

**Affiliations:** 1 Division of Surgical Oncology, Department of Surgery, University of California, San Francisco, 505 Parnassus Ave, S549, Box 1932, San Francisco, California 94143-1932 USA; 2 Division of Hematology and Oncology, Department of Medicine, University of California, San Francisco, San Francisco, California USA

**Keywords:** Melanoma, Metastasectomy, Small Bowel Resection, Immunotherapy, Checkpoint Inhibitors

## Abstract

**Background:**

Metastatic melanoma to the small bowel is an aggressive disease often accompanied by obstruction, abdominal pain, and gastrointestinal bleeding. With advancements in melanoma treatment, the role for metastasectomy continues to evolve. Inclusion of novel immunotherapeutic agents, such as checkpoint inhibitors, into standard treatment regimens presents potential survival benefits for patients receiving metastasectomy.

**Case Presentation:**

We report an institutional experience of 15 patients (12 male, 3 female) between 2014-2022 that underwent small bowel metastasectomy for metastatic melanoma and received perioperative systemic treatment. Median age of patients was 64 years (range: 35-83 years). No patients died within 30 days of their surgery, and the median hospital length of stay was 5 days. Median overall survival in these patients was 30.1 months (range: 2-115 months). Five patients died from disease (67 days, 252 days, 426 days, 572 days, 692 days postoperatively), one patient died of non-disease related causes (1312 days postoperatively), six patients are alive with disease, and three remain disease free.

**Conclusions:**

This case series presents an updated perspective of the utility of metastasectomy for small bowel metastasis in the age of novel immunotherapeutic agents as standard systemic treatment. Small bowel metastasectomy for advanced melanoma performed in conjunction with perioperative systemic therapy is safe and appears to promote long-term survival and enhanced quality of life.

## Introduction

Metastatic cutaneous melanoma remains a highly aggressive disease that possesses a dismal prognosis despite recent efforts to expand the standard regimen of treatment. Small bowel involvement of advanced melanoma is rare though well-described, and harbors grave repercussions including obstruction, abdominal pain, and chronic gastrointestinal bleeding. While surgery presents curative potential for patients with Stage I-IIIB disease, the role for metastasectomy in metastatic melanoma continues to be defined given advancements in melanoma treatment. For patients with stage IV melanoma, surgical resection of metastases may be accompanied by immunotherapy, targeted therapy, radiation, and chemotherapy [1]. Efforts to evaluate chemotherapeutic combinations have shown a lack of significant improvement in overall survival [2], and efficacy of targeted therapies requires patients to possess mutations in key signaling pathway genes [1].

Various randomized, controlled trials have compared response rates and long-term oncologic outcomes between immune checkpoint inhibitors and cytotoxic chemotherapy. The CheckMate 066 trial demonstrated significant benefits in overall survival for previously untreated metastatic melanoma patients treated with nivolumab monotherapy compared to dacarbazine treatment, and these findings were subsequently confirmed on 5-year analysis [3, 4]. Similarly, BRAF V600E-positive mutation metastatic melanoma patients treated with targeted therapies (BRAF/MEK inhibitors) have shown superior progression-free survival compared to those treated with cytotoxic chemotherapy in the BREAK-3 (dabrafenib versus dacarbazine )[5, 6] and METRIC trials (trametinib versus various chemotherapies )[7, 8]. Given the advent of novel immunotherapeutic agents for advanced melanoma, single-agent or combination treatments of checkpoint blockade agents, such as anti-CTLA-4 and anti-PD-1/PD-L1 antibodies, have gained traction as preferred immunotherapeutic treatment options because of their substantial efficacy in prolonging overall survival.

Thus, patients receiving surgical resection of metastases may experience enhanced prognoses from systemic treatments in both the neoadjuvant and adjuvant settings [9]. Here, we report 15 patients that underwent small bowel metastasectomy for advanced melanoma and received perioperative systemic immunotherapy or targeted treatment. We hypothesized that small bowel resection for metastatic melanoma is associated with improved outcomes for patients receiving these therapies.

## Case Presentation

**Fig. 1:**
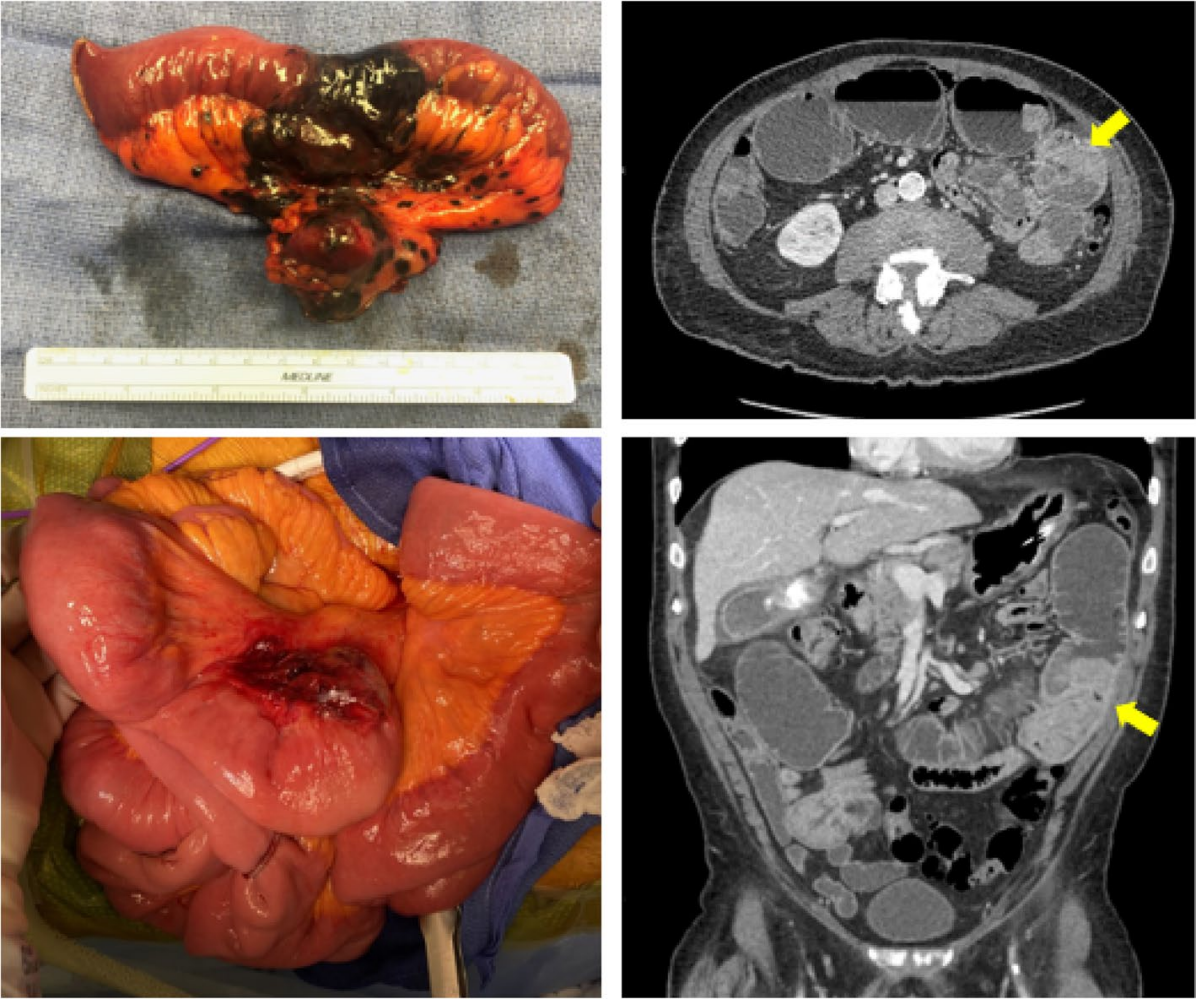
Left panels: intraoperative examples of metastatic melanoma lesions to small bowel. The upper left panel demonstrating melanosis, while the lower left depicts mesenteric invasion. Right panels: axial and coronal CT images of intraluminal metastatic melanoma causing obstruction

Institutional medical records were reviewed for patients with metastatic melanoma to the small bowel that underwent small bowel resection between 2014-2022. Fifteen consecutive patients (12 male, 3 female) were identified to have received small bowel resection for metastatic melanoma (Figure 1). The median age and standard deviation of the patients was 63.7±13.7 years old and the range was 35-83 years. The median time interval that occurred from index diagnosis of melanoma to detection of small bowel metastasis was 55.1 months (range: 0-207 months), with a median interval until small bowel resection of 113.3 months (range: 1-226 months). Table 1 describes demographic, clinicopathologic, and postoperative outcomes for the patients in this case series. 11 patients received neoadjuvant therapy prior to small bowel resection, including ipilimumab/nivolumab (9), BRAF/MEK inhibitor (5), pembrolizumab (3). 14 patients received adjuvant therapy after small bowel resection, including ipilimumab/nivolumab (2), pembrolizumab (4), BRAF/MEK inhibitor (4), and nivolumab (3).

**Table 1:** Demographic, clinicopathologic, and postoperative outcomes of patients with Stage IV melanoma to the small bowel who underwent metastasectomy

Patient ID	Gender	Age at First Small Bowel Resection	Location of Primary Melanoma	BRAF V600E Mutation Status	Time from Melanoma Dx to First SB Resection (years)	Sites of Additional Metastasis	Systemic Therapy Prior to SB Resection	Indication for Small Bowel Resection	Number of Bowel Segments Resected	Pallative or Curative Intent	Adjuvant Therapy	Post-operative Follow-up Duration (months)	Patient Status
1	Male	69	Unknown	Wild-type	0.2	Lung and pancreas	Nivolumab	Intussusception	2	Palliative	Nivolumab	23.7	DOD
2	Male	75	Lower extremity	Wild-type	25.5	None	None	GI bleeding	1	Curative	Pembrolizumab	43.2	DUD
3	Male	83	Unknown	Wild-type	1.5	Stomach, lungs, liver, femur, mesentery	Ipilimumab + Nivolumab	Sub-acute obstruction	1	Curative	Nivolumab	53.8	AWD
4	Male	60	Neck	Wild-type	2.7	Liver	Ipilimumab + Nivolumab, IL-12	GI bleeding	3	Palliative	PD-1 + PI3K inhibitor trial	13.6	DOD
5	Female	35	Hip	Mutant	15.2	Brain and lungs	BRAF/MEK inhibitor, Pembrolizumab, Ipilimumab + Nivolumab	Obstruction	1	Palliative	BRAF/MEK inhibitor	47.5	AWD
6	Male	74	Uvea	Wild-type	18.8	Brain, liver, pancreas, omentum	Ipilimumab + Nivolumab	Elective debulking	1	Palliative	Abexinostat + Pembrolizumab trial	18.8	DOD
7	Male	37	Scalp	Mutant	1.6	Peri-auricular, spleen, kidney	BRAF/MEK inhibitor, Pembrolizumab + PV-10 trial, abexinostat + pembrolizumab trial	GI bleeding, obstruction	4	Palliative	None	2.2	DOD
8	Female	67	Upper extremity	Mutant	0.1	Brain	BRAF/MEK inhibitor, Ipilimumab + Nivolumab	Perforation	1	Curative	BRAF/MEK inhibitor	82.6	NED
9	Male	55	Chest	Wild-type	17.2	Lungs and peritoneum	None	GI bleeding	1	Curative	Ipilimumab, Pembrolizumab	114.7	NED
10	Male	58	Toe	Wild-type	0.1	Lung and mesentery	None	Intussusception	2	Palliative	Ipilimumab + Nivolumab	30.1	AWD
11	Male	67	Scalp	N/A	9.5	Lung	Ipilimumab + Nivolumab	Obstruction, GI bleeding	4	Palliative	Chemotherapy	26.3	AWD
12	Female	79	Scalp	Wild-type	9.4	Liver, pancreas, lungs	None	Elective debulking	1	Curative	Pembrolizumab, Ipilimumab + Nivolumab	40.2	AWD
13	Male	61	Unknown	Mutant	1.8	Liver, peritoneum, adrenal glands	BRAF/MEK inhibitor, Ipilimumab + Nivolumab, Verzenio	Elective debulking	1	Palliative	BRAF/MEK inhibitor	30.1	AWD
14	Male	73	Right abdomen	Wild-type	10.2	Brain	Pembrolizumab, Ipilimumab + Nivolumab, MEK inhibitor	GI bleeding	4	Palliative	MEK inhibitor	8.4	DOD
15	Male	62	Right upper back	Mutant	14.3	Mesentery	Ipilimumab + Nivolumab	Abdominal pain	1	Curative	Nivolumab	23.1	NED

*AWD* alive with disease, *DOD* died of disease, *DUD* died unrelated to disease, *Dx* diagnosis, *BRAF* proto-oncogene B-Raf, *MEK* mitogen-activated protein kinase kinase, *PD*-1 programmed cell death protein 1, *PI3K* phosphoinositide 3-kinase, *GI* gastrointestinal, *SB* small bowel, *Abexinostat* histone deacetylase (HDAC) inhibitor, *Verzenio* cyclin-dependent kinase (CDK) inhibitor

Five patients (33%) possessed BRAF V600E mutations. The main indications for surgical resection included perforation (1), subacute obstructive symptoms (1), gastrointestinal bleeding (6), acute obstruction/intussusception (5), abdominal pain (1), and asymptomatic/detection on surveillance imaging (3). Palliative resection was indicated for nine patients, whereas six patients underwent curative resection for small bowel metastases. Median bowel length resected was 9 cm (range: 3-152 cm) with metastasis size of 5 cm (range: 2-15 cm). The median number of small bowel segments resected was 1 (range: 1-4). For all cases, primary anastomosis was performed; no stomas were required. No patients died within 30 days of their surgery, and the median hospital length of stay was 5 days. Survival was defined as the time interval between the patient’s metastasectomy and death of the patient or last follow-up. Median overall survival for all patients was 30.1 months (range: 2-115 months) and was 48.6 months and 23.4 months for patients that underwent curative and palliative resections, respectively. Five patients died from disease (67 days, 252 days, 426 days, 572 days, 692 days postoperatively), one patient died of non-disease related causes (1312 days postoperatively), six patients are alive with disease, and three remain disease free.

## Discussion

Metastatic melanoma to the small bowel is a disease traditionally associated with dismal prognosis and is accompanied by substantial adverse events, such as obstruction, abdominal pain, perforation and chronic GI bleeding. In the context of these symptomatic indications, small bowel metastasectomy is often performed. This series provides the accounts of 15 patients treated at our institution who underwent small bowel resection for metastatic melanoma and received perioperative immunotherapy or targeted therapy. To our knowledge, this is largest case series of patients who underwent surgical resection for GI melanoma metastases that all received perioperative immunotherapy and presents an updated perspective of the utility of metastasectomy for small bowel metastasis in the age of novel immunotherapeutic agents as standard systemic treatment.

Most notably, the median survival following operative intervention observed in this case series (overall 30.1 months, curative 48.5 months, palliative 23.7 months) greatly surpasses those reported in prior studies concerning patients receiving surgical resection of metastatic melanoma to the gastrointestinal tract [10–12]. In a cohort of 68 patients with metastatic melanoma to the GI tract, Agrawal et al. cited a median overall survival of 8.2 months, with the majority of patients (61.4%) treated in the adjuvant setting receiving chemotherapy [10]. Similarly, Mantas et al. reported a median overall survival of 14 months in patients with metastatic melanoma to visceral surfaces that all had received neoadjuvant palliative chemotherapy. Lastly, an investigation by Sanki et al. of 117 patients who underwent surgical resection for GI melanoma metastases yielded a median overall survival of 16.4 months, but adjuvant therapy was only administered to a portion of patients (chemotherapy: 29.1%, immunotherapy: 17.1% )[12]. The survival differences between this present case series and prior reports is that these studies were conducted before the implementation of immunotherapies as standard treatment regimen, whereas the timeframe of our patients encompasses the efficacy of checkpoint inhibitors in the context of metastatic melanoma.

Surgical resection for stage IV melanoma to the small bowel continues to be debated, as some have reported that elective surgery can improve quality of life but does not confer to survival benefits [11]. Conversely, proponents for surgery have found that metastasectomy is an independent predictor of survival compared to patients who were ineligible for surgery and others managed conservatively [11, 12]. These findings were less consistent for patients undergoing palliative-intent resections, as there still remains questions about whether surgery can prolong overall survival in addition to improvements in quality of life [12].

However, the utility of small bowel metastasectomy has not been previously investigated in the setting of immune checkpoint inhibitors. In this case series, all patients received checkpoint blockade therapy, with the most common being anti-PD-1 and anti-CTLA-4 inhibitors, or a combination of both. This represents a shift in the standard of treatment for metastatic melanoma from chemotherapies, like Dacarbazine, to immunotherapeutic options. The efficacy of immunotherapies, namely immune checkpoint inhibitors, in advanced melanoma furthers the rationale for pursuing metastasectomy, especially since the surgery can be performed with low morbidity and mortality [10], as seen in our case series.

Overall, our study demonstrates that small bowel resection for well selected patients with metastatic melanoma in the era of immune systemic therapy is safe and appears to promote long-term survival and enhanced quality of life. Thus, small bowel metastasectomy should be offered to appropriately selected Stage IV melanoma patients in this setting.

## Data Availability

The datasets used and/or analysed during the current study are available from the corresponding author on reasonable request.

## Abbreviations

CTLA-4: Cytotoxic T lymphocyte-associated antigen
PD-1: Programmed cell death protein 1
PD-L1: Programmed death-ligand 1
BRAF: v-raf murine sarcoma viral oncogene homolog B1
MEK: Mitogen-activated extracellular signal-regulated kinase
GI: Gastrointestinal
